# Alteration of mRNA abundance, oxidation products and antioxidant enzyme activities during oocyte ageing in common carp *Cyprinus carpio*

**DOI:** 10.1371/journal.pone.0212694

**Published:** 2019-02-22

**Authors:** Azin Mohagheghi Samarin, Azadeh Mohagheghi Samarin, Tone-Kari Knutsdatter Østbye, Bente Ruyter, Sabine Sampels, Viktoriia Burkina, Miroslav Blecha, David Gela, Tomas Policar

**Affiliations:** 1 University of South Bohemia in Ceske Budejovice, Faculty of Fisheries and Protection of Waters, South Bohemian Research Center of Aquaculture and Biodiversity of Hydrocenoses, Research Institute of Fish Culture and Hydrobiology, Vodňany, Czech Republic; 2 Nofima (Norwegian Institute of Food, Fisheries and Aquaculture Research), Ås, Norway; 3 Department of Molecular Sciences, Swedish University of Agricultural Sciences, Uppsala, Sweden; Tokat Gaziosmanpasa University, TURKEY

## Abstract

Oocyte ageing is the most important factor affecting egg quality of several fish species after ovulation. Oxidative stress has been proposed as the initiator of the oocyte ageing process in other vertebrates. To identify the role of oxidative stress and apoptosis on the progress of oocyte ageing in the common carp *Cyprinus carpio*, changes in the relative mRNA abundance of selected transcripts were examined. The possible alteration in the oxidation status of the oocytes during ageing was also studied. In addition, the activity of antioxidant enzymes during oocyte ageing was evaluated. Oocytes from 6 females were incubated *in vivo* for 14 hours post-ovulation (HPO) and *in vitro* for 10 hours post-stripping (HPS) at 20°C before fertilization. Hatching rates were over 65% up to 4–6 HPO, finally dropping to 1.3% at 12–14 HPO.Hatching rates were over 65% up to 4–6 HPO, finally dropping to 1.3% at 12–14 HPO. Hatching rates were more than 70% for the eggs stored *in vitro* up to 6 HPS and then decreased to 21.3% at 10 HPS. The results demonstrated no significant changes in the relative mRNA levels of oxidative stress-related genes or genes involved in the cell cycle during the progress of oocyte ageing in common carp. Additionally, the amount of TBARS and carbonyls did not change as time elapsed following ovulation. The apoptosis-related genes however, were significantly altered following the prolonged time interval between ovulation and fertilization. The lack of response of both activities of antioxidant enzymes and oxidation products during oocyte ageing strengthens the conclusion that oxidative stress is unlikely to be a main factor determining the progress of oocyte ageing in common carp. However, an increase in the mRNA abundance of apoptosis-related genes demonstrates that apoptotic pathway might be involved in the progress of oocyte ageing.

## Introduction

Fertilization success, embryo quality and later life of the offspring are highly dependent on the integrity of the oocyte, which contains important information for orchestrating embryogenesis [1, 2] and for remodelling parental genomes [3, 4]. Oocyte ageing, which refers to the time period between ovulation and fertilization, has been identified as the most important factor affecting egg quality of several fish species after ovulation [5–7]. During ovulation, mature eggs are released from follicle cells into the ovarian or body cavity while they are still in metaphase of the second meiotic division stage [8]. After ovulation, the eggs remain in the ovarian or body cavity until the occurrence of spawning, which is stimulated by environmental factors, or until the eggs are collected by artificial techniques. Delayed spawning in nature, delayed egg stripping in culture conditions and delayed fertilization (after egg collection) lead to oocyte ageing, and finally, egg over-ripening. During oocyte ageing, major morphological, physiological, biochemical, histological, cellular and molecular changes occur inside the eggs [9]. These changes deteriorate the quality of ovulated eggs and lead to a limited fertilization rate [10, 11], increased larval malformation [12–14] and increased ploidy anomalies [15, 16]. The successful egg storage time time period during which eggs remain viable after ovulation and stripping varies from a few minutes to a few weeks depending on the fish species and storage temperature [9].

Oocytes are large cells responsible for embryo development by providing the embryo with transcripts and proteins until the onset of zygotic transcription. Therefore, the dependence of the early stages of embryogenesis on maternal mRNAs and proteins are not surprising [17]. The effect of fish oocyte ageing on several aspects of egg quality has been studied. However, until now, there has been only poor understanding about the processes and underlying mechanism of oocyte ageing in fish, as well as other vertebrates. There are few studies analysing the genomics and transcriptomics of egg quality associated with the oocyte ageing in fish. Different quantities of mRNAs between over-ripened and freshly ovulated rainbow trout *Oncorhynchus mykiss* eggs have been reported [18–20]. In Atlantic halibut *Hippoglossus hippoglossus*, poor hatching success was correlated with low transcript levels of specific genes [21]. In rainbow trout eggs, ova ageing resulted in the downregulated expression of specific microRNAs and their target genes mainly involved in cell death and signal transduction, stress response and DNA damage, RNA degradation, and energy and transcription regulation [22].

Studies on other vertebrates have proposed the involvement of oxidative stress as the initiating factor on the progress of oocyte ageing [23–25]. These studies report that oxidative stress can, in turn, trigger many cascades affecting oocyte quality, such as mitochondrial dysfunction, DNA damage, perturbed Ca^2+^ homeostasis and lipid peroxidation. In general, ageing is associated with increases in levels of endogenous reactive oxygen species (ROS) and decreases in antioxidant defences; leading to a wide range of oxidative damage in cell structures, including lipid peroxidation of membranes, enzyme inactivation, protein oxidation, and DNA damage [26–28]. Alteration of the lipidome, associated with oocyte ageing, was evaluated in a mouse model [29]. The latter study reported that several phospholipid classes were significantly decreased in aged oocytes, which suggests the involvement of oxidative damage in lipid plasma membrane composition and, as a result, unfavourable outcomes of oocyte ageing. The enzymatic antioxidant system can scavenge ROS and therefore decrease the effect of oxidative stress. Very little information is available about how oocyte antioxidant defences change during oocyte ageing. Additionally, a decline in critical cell cycle factors [30] and impaired mitochondrial function [31, 32] have been shown be related to the deleterious effects of oocyte ageing.

Fish are good model animals to evaluate oocyte ageing because they display a vast diversity of reproductive modes, and most species produce a high number of oocytes compared with that of other animals. Due to some ethical concerns and the intrinsic nature of other vertebrates’ oocytes, it is difficult to study the oocyte ageing process. Furthermore, as the demand for assisted reproduction technology is increasing and very few studies in this field are available, a comparative study on the process of oocyte ageing in fish might be beneficial for research on other vertebrates. The present study examines some cellular and molecular changes associated with oocyte ageing in the common carp *Cyprinus carpio*, focusing on the possible role of oxidative stress on the progress of the time-dependent oocyte over-ripening process. The evaluation was done at the levels of transcriptome and antioxidant enzyme assays, lipid and protein oxidation status as well as the egg phenotype and functional changes during the oocyte ageing. The common carp was selected for the experiment because the ovulation of the eggs occurs simultaneously in each individual female and our previous experience with the oocyte ageing in this species was satisfactory regarding practical approaches [11]. Genes involved in oxidative damage and stress response, mitochondrial function, fertilization, embryo development, transcriptional regulation and cell cycling as well as the ones related to the apoptosis were screened for their mRNA abundance during egg over-ripening. We also investigated the alteration in oxidation status of oocytes during post-ovulatory ageing by measuring thiobarbituric acid reactive substances **(**TBARS) as a marker of lipid oxidation, and carbonyls, which show the extent of protein oxidation. In addition, the role of oxidative stress in the progress of oocyte ageing was assessed by evaluation the activity of antioxidant enzymes, catalase (CAT), superoxide dismutase (SOD), glutathione peroxidase (GPX) and glutathione reductase GR, were examined during oocyte ageing. The combination of the aforementioned parameters will give a broad picture and understanding of the ongoing mechanisms in the oocyte ageing process. Identifying molecular mechanisms involved in the decline of oocyte quality with the progress of oocyte ageing could have important implications for aspects of basic research and practically applications for aquaculture purposes to prevent or delay oocyte ageing.

## Materials and methods

### Ethics

The experimental procedures were performed according to the ethical rules of the EU-harmonized Animal Welfare Act of the Czech Republic. The unit is licensed (No. 53100/2013-MZE-17214) according to the Czech National Directive (the Law against Animal Cruelty, No. 246/1992). The methodological protocol of the current study, experimental manipulations and sampling procedures were specifically approved by the expert committee of the Institutional Animal Care and Use Committee (IACUC) of the University of South Bohemia, Czech Republic. Two co-authors of this study involved in the field part of the experiment own the certificates (CZ 01672 and CZ 01660) giving permission to conduct and manage experiments involving animals according to section 15d paragraph 3 of Act no. 246/1992 Coll. The study did not involve endangered or protected species. To examine ovulation and to collect gametes, brood fish were anaesthetized with a 0.03 ml/L clove oil water bath to enhance animal welfare and minimize stress.

The study was carried out at the Genetic Fisheries Center (GFC), Research Centre of Aquaculture and Biodiversity of Hydrocenoses (RIFCH), University of South Bohemia (USB), Vodnany, Czech Republic with geographic coordinates of 49.1479° N, 14.1751° E. The permission to conduct the study was issued by the owner of the site; RIFCH, USB.

### Fish

Brood fish were captured from earthen ponds when the average daily water temperature reached 16°C during the breeding season at the Genetic Fisheries Center (GFC), Research Centre of Aquaculture and Biodiversity of Hydrocenoses (RIFCH), University of South Bohemia (USB), Vodnany, Czech Republic with geographic coordinates of 49.1479° N, 14.1751° E. The fish were then transferred to indoor rectangular-shaped tanks (each 5.7 m^3^ capacity) supplied with water from a recirculating system and maintained under dim conditions (<20 lx), with females and males housed separately. The holding temperature was gradually increased to 20°C over time. After the experiment, broodfish were housed back to the earthen ponds at the GFC, RIFCH, USB for the purpose of using in the future research. The larvae originating in the study also were released to the small earthen ponds center. None of the experimental animals either broodfish or the larvae were sacrificed during this study.

### Egg storage in the ovarian fluid and gamete collection

After a 3-day period of acclimation, female brood fish were treated with an intramuscular injection of carp pituitary homogenate CPH (0.3 mg kg^-1^ body weight). This step was followed by a second injection with CPH (3.5 mg kg^-1^ body weight) 12 hours later. Male brood fish (4.5 ± 0.4 kg body weight, mean ± SEM) were subjected to a single intramuscular injection of CPH (2 mg kg^-1^ body weight) that was given simultaneously with the second injection of the females. These procedures were performed according to Horvath and Tamas, 1985 [33]. The females were examined for ovulation every 2 hours, starting 10 hours after the second injection. Six females that ovulated within 2 hours (weighing 4.2 ± 0.2 kg body weight), were selected randomly, marked with coloured tags and used for the experiment. Almost half of the eggs were stripped from each female and used for the *in vitro* egg storage experiment. The other half of the eggs was retained inside the fish bodies to be used for the *in vivo* egg storage experiment. These two experiments were performed as described below.

### *In vivo* oocyte ageing

In total, seven batches of eggs from each of the six females were separately stripped within 2- hour intervals and then fertilized during the experimental period of 14 hours after ovulation. An egg batch of 7 g was stripped individually from each female and fertilized immediately (0–2 hours post-ovulation, HPO). The rest of the eggs were left inside the ovarian cavity of each female for the next fertilization time. Thus, eggs were retained inside the fish body before fertilization for 0–2, 2–4, 4–6, 6–8, 8–10, 10–12 and 12–14 HPO.

### *In vitro* oocyte ageing

Seven batches of 7 g egg aliquots were collected from each of the six females and stored in sterile six-well cell culture plates (each well diameter: 3.5 cm). The eggs were stored two layers deep [34] in the ovarian fluid, and no solution, artificial media or extender was added. All plates were individually covered by their own lids and stored in the dark at 20°C in the laboratory incubator for 10 hours. To provide the humidified atmosphere, a few plates were filled with water and placed into the storage chambers [34, 35]. Stored ova were fertilized at 0 (immediately after stripping), 2, 4, 6, 8, 10 and 12 hours post-stripping.

### Artificial fertilization and egg development

For each sampling time, egg quality was evaluated by measurement of the eyeing, eyed-egg mortality, hatching and larval malformation rates. Artificial fertilization, incubation and examination of egg developmental success were performed according to Samarin et al. [11].

### RNA isolation and reverse transcription

At all HPOs and HPSs, 1 g of the unfertilized eggs was placed in cryotubes, frozen in liquid nitrogen and then stored at -80°C freezer until RNA isolation. Samples were collected in three replicates for each fertilization time.

To prepare total RNA, an equal weight of 20 eggs was taken from each tube and treated with TRIzol reagent (Invitrogen Corporation, Carlsbad, CA) in Precellys24 homogenizer (Bertin Instruments), 5500 rpm for 2×20 sec with a 5 sec interval. RNA was isolated using the PureLink RNA Purification Kit (Invitrogen) according to the manufacturer’s instructions. During RNA isolation, samples were depleted of genomic DNA using a PureLink DNase Kit (Invitrogen) according to the manufacturer’s protocol. The RNA concentration and RNA purity were assessed using a NanoDrop Spectrophotometer (ND-1000, Thermo Scientific).

A concentration of 1000 ng RNA was used to generate cDNA using TaqMan reverse transcription Reagents (Life Technologies). Random hexamers were used to prime the reaction. Reverse transcription was performed at 25°C for 10 min, at 48°C for 1 hour and finally 95°C for 5 min. Control reactions were run without TaqMan reverse transcriptase and used as negative controls in the real-time PCR study.

### Real-time PCR analysis

Nucleotide sequences corresponding to common carp mRNA (NCBI) were used for primer design (by Primer3) [36, 37]. Transcript levels of 16 genes, *hsp70*, *cox1*, *sod*, *gpx1*, *cyclinA*, *cyclinB*, *jnkA*, *jnkB*, *caspase3A*, *caspase9*, *bax*, *bcl2*, *cathepsinB*, *cathepsinZ*, *vasa* and *igf2*, were determined by real-time PCR in duplicate using the LightCycler 480 (Roche Applied Science, Germany). The reaction mix for real-time PCR consisted of 4 μl diluted (1:10) cDNA, 1 μl forward and reverse primer (final concentration of 500 nM, Table 1), and 5 μl SYBR Green-I Master (Roche). All primers were provided by Invitrogen. A standard curve was included for each primer pair to evaluate primer efficiency. All samples were analysed in parallel, and a non-template control with water substituted for cDNA was run for each primer pair. The real-time PCR reaction was run under the following conditions: preincubation at 95°C for 5 minutes, amplification for 45 cycles at 95°C for 15 seconds and 60°C for 1 minutes, melting curve at 95°C for 5 seconds and 65°C for 1 minutes, cooling at 40°C for 10 seconds.

**Table 1:** Real-Time PCR primer sequences.

*Target gene*	*Forward primer (5′-3′)*	*Reverse primer (5′-3′)*	*Genbank accession no*.
*hsp 70*	GAGAGGCTGATTGGAGATGC	ACTGCACAACTGGGTCATCA	HQ259767
*cytochrome c oxidase I*	TCCACGGAGGATCCATTAAA	GGATAGGACGATCCCTGTGA	HQ235998
*vasa*	AGGCCAGGAAGTTTGCCTAT	TGCAGCCCTTTAACACCTCT	KP661178
*Caspase3a*	TGATGGCAAAGTATGGCAAA	ATCAAAGACTGGCTGGTTGG	KF055462
*Caspase9*	CCTGTGGAGGAGGTGAGAAG	ATGGGAATAGCGTCCATCTG	KC676314
*18s ribosomal RNA*	AGTTCCGACCGTAAACGATG	AGACTCGTGGTTTCCCACAC	JQ619778
*beta-actin gene*	GCAAAGGTTTTGTGCTCCAT	CATGGATACCGCAAGATTCC	JQ619775
*Bcl2*	GGGATGCCTTTGTGGAGTTA	TCACTCCTGCCAAGCCTAGT	EU490408
*Bcl2 associated X protein (Bax)*	GGAGATGAGCTGGATGGAAA	AAGATCTCTCGGGCCACTTT	KJ174685
*glutathione peroxidase (Gpx1)*	GGAGAAGCTGGAAGTGAACG	TCACCCATCAAGGACACAGA	GQ376155
*gapdh*	GTGATGCAGGGGCTCAGTAT	CTCTCTTGGCACCACCCTTA	AJ870982
*IGF2*	TGCAAAACCCATGAAGTCTG	AAGAGGCCCTCCTGAGATGT	KP663718
*cyclin A*	TGCATGTCTGTCCTGAGAGG	TCCACTTCCGGAGGATACAC	EU380205
*cathepsin Z*	GAGAGAAAGGCTGGCTCAGA	GGGTCTCCGTACATGCAGTT	AY949988
*cathepsin B*	AAAGGACCCAGACTCCCTGT	TTTAAGAGTGGGGCAGTTGG	AB215097
*Mn/ SOD*	TTATGCAGCTTCACCACAGC	ACATCACCCTTAGCCAGTGC	JF411603
*cyclin B*	CCAGAAAAGCAGCTGTAGCC	TCTTCCTCAAAGCCTGTCGT	EU293852
*jnkA*	TCGATGAGAGGGAACACACA	GACCTCGAATGACACCGTTT	JN542470
*jnkB*	CCAACCTCTGCCAAGTCATT	CCGAGTGGAGGTGTTTTGTT	AB001744

The comparative CT method was used for relative quantification of target gene expression levels. To normalize relative gene expressions, three reference genes, glyceraldehyde 3-phosphate dehydrogenase (*gapdh*), 18S ribosomal RNA (*18s*) and beta-actin (*b-actin*), were tested. The mRNA abundance of *gapdh* proved to be highly stable in common carp eggs collected at different time points after ovulation, and it did not show any significant change in Ct values in the eggs at different HPOs and HPSs. Therefore, *gapdh* proved the most stable and was used as the reference gene. Relative expression was then calculated according to the equation 2^**-Δ**Ct^. Sequences of primers used are listed in Table 1.

### Antioxidant enzyme activity assays

#### Preparation of post-mitochondrial supernatant

Samples of fish eggs (approx. 400 mg) were homogenized using a Tissue Lyser II (Qiagen) in 0.1 M K-phosphate buffer (pH 7.4). Homogenates were centrifuged at 15,000 g, 4°C for 15 min in a Micro 200 R. Supernatants were removed, stored at 0–4°C and used for protein determination and enzyme assays. Protein levels were measured spectrophotometrically using bovine serum albumin as a standard [38]. The samples were diluted to a protein content of 10 mg/mL.

#### Antioxidant enzyme activity

Catalase (CAT) activity was quantified as a decrease in hydrogen peroxide in a 96-well flat-bottomed UV-transparent microtitre plate. CAT activity was assessed spectrophotometrically at 240 nm and was performed following the method of Claiborne, 1985 [39]. Calculations were made using a molar extinction coefficient of 40 M^−1^ cm^−1^.

The superoxide dismutase (SOD) activity was determined by the method of Nishikimi et al. [40]. Superoxide dismutase activity was assessed spectrophotometrically at 420 nm and expressed as the nitro blue tetrazolium formation per milligram of protein per minute.

Glutathione peroxidase (GPX) activity was measured using the method of Mohandas et al. [41]. Briefly, incubation mixture (0.3 mL) contained post-mitochondrial supernatant (approx. 0.2 mg of protein), K-phosphate buffer 0.05 M (pH 7.0), in EDTA 1 mM, sodium azide 1 mM and glutathione reductase from baker's yeast (7.5 ml from stock containing 1 U/ml), reduced glutathione 4 mM and NADPH 0.8 mM. The reaction was started via the addition of 0.5 mM of hydrogen peroxide. The oxidation of NADPH was recorded at 340 nm. Calculations were made using the molar extinction coefficient 6220 M^−1^ cm^−1^.

The glutathione reductase (GR) activity was measured by the method of Cribb et al. [42]. Briefly, incubation mixture (0.25 mL) contained post-mitochondrial supernatant (approx. 0.3 mg of protein), K-phosphate buffer 0.05 M (pH 7.0), NADPH (0.4 mM), oxidized glutathione (0.4 mM) and diethylenetriaminepentaacetic acid (DTPA) (1 mM). Disappearance of NADPH was measured at 340 nm and calculated as NADPH oxidized formation using the molar extinction coefficient of 6220 M^−1^ cm^−1^.

All assays were performed spectrophotometrically in quadruplicate or triplicate using a 96-well microplate reader (Tecan Infinite M200, Germany). Samples were held on ice; measurements were made at 25°C. Variations in absorbance at each reaction well were linear over time (R^2^>0.8).

#### Thiobarbituric acid reactive substances

The thiobarbituric acid reactive substances **(**TBARS) method was used to evaluate oocyte lipid peroxidation according to the method described by Li et al. [43]. After reaction with thiobarbituric acid in darkness for 15–20 hours (overnight) at room temperature (20°C), the reaction complex was detected at a wavelength of 530 nm against the sample blank using a UV-visual plate reader (AF 2200; Austria). The results were expressed as equivalents to malonaldehyde (MDA) in μg/g.

#### Protein oxidation

Protein oxidation was estimated as carbonyls after incubation with 2,4-dinitrophenylhydrazine (DNPH) in 2 N hydrochloric acid following a slightly modified method from the one described by Oliver et al. [44]. Carbonyl concentration was analysed as DNPH calculated on the basis of absorption of 21.0 mM-1 cm-1 at 370 nm for aliphatic hydrazones. The protein concentration was measured at 280 nm in the same sample and quantified using bovine serum albumin as a standard.

### Statistical analysis

The normality of the data was ascertained using SPSS software version 18. Differences between the means of the groups for each measured egg quality parameter (eyeing, hatching, eyed-egg mortality and larval malformation rates) as well as each individual gene, lipid and protein oxidation levels and antioxidant enzyme activity were evaluated using an ANOVA followed by Duncan’s multiple range test. Wilcox test; a non-parametric statistical hypothesis test, was used to compare the *in vivo* and *in vitro* egg storage results. *P < 0*.*05* was considered to be significant.

## Results

### Egg viability

#### *In vivo* oocyte ageing

**Fig 1:**
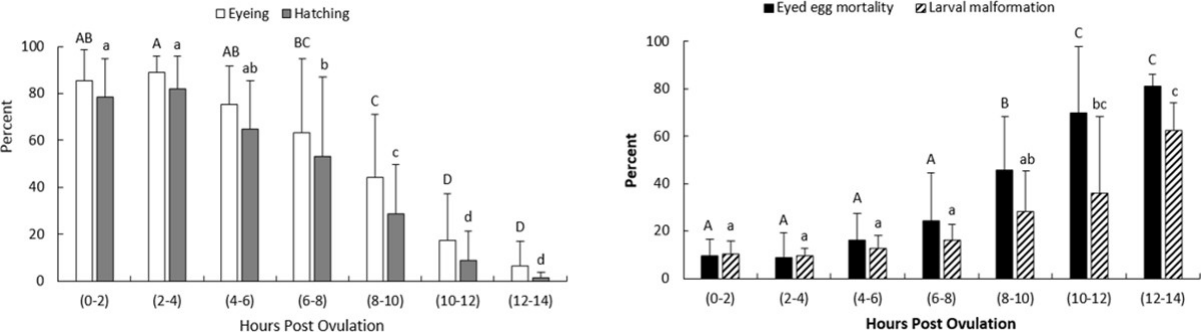
Effects of *in vivo* oocyte ageing on the eyeing, hatching, eyed-egg mortality and larval malformation rates in common carp (mean ± SD). Means sharing a common alphabetical symbol do not differ significantly.

The eyeing and hatching values remained almost constant, approximately 83% and 75%, respectively, for the eggs fertilized up to 4 HPO (Fig 1). Although not significantly different, the eyeing and hatching rates increased to 89% and 82%, respectively, at 2–4 HPO. Thereafter, the values decreased linearly over time and dropped to 6.3% for the eyeing rates and 1.3% for the hatching rates at 12–14 HPO. Eyed-egg mortality and larval malformation rates were not significantly different for the eggs fertilized up to 6 hours after ovulation. The highest eyed-egg mortality and larval malformation were found at 12–14 HPO, 80.9 ± 5.1% and 62.5 ± 11.8%, respectively.

#### *In vitro* oocyte ageing

**Fig 2:**
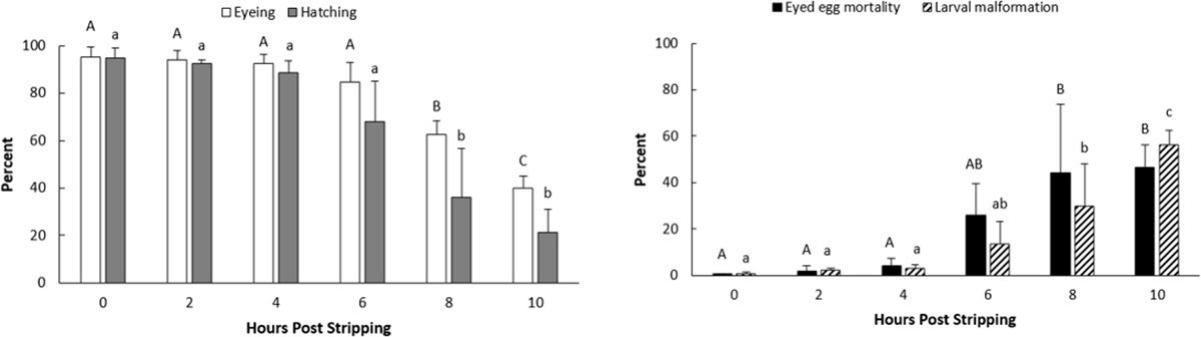
Effects of *in vitro* oocyte ageing on the eyeing, hatching, eyed-egg mortality and larval malformation rates in common carp (mean ± SD).

The eyeing and hatching rates were almost constant, approximately 91% and 86%, respectively, up to 6 HPS (Fig 2). After 10 HPS the eyeing and hatching rates were measured to be 40 ± 5.1% and 21.3 ± 9.7%, respectively. The eyed-egg mortality and larval malformation rates also did not show any marked increase during 6 hours of the *in vitro* egg storage. After 6 HPS, the eyed-egg mortality and larval malformations increased significantly over time so that at 10 HPS, 46% of the eyed-eggs died, and 56% of the hatched larvae were malformed.

### Relative mRNA abundance of selected transripts

#### *In vivo* oocyte ageing

**Fig 3:**
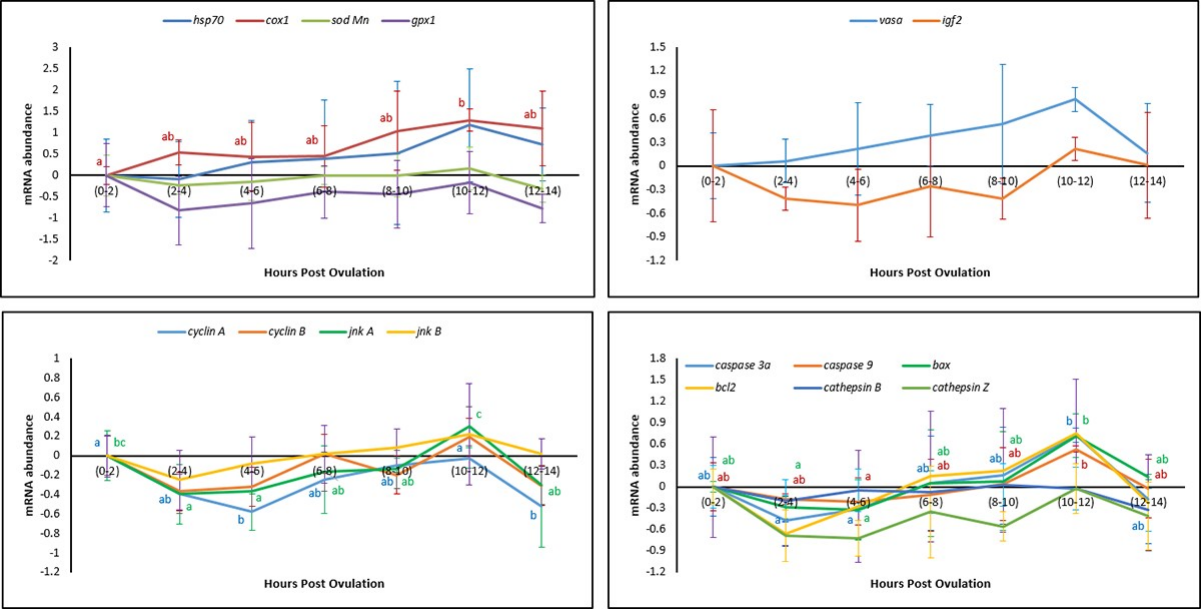
Effect of *in vivo* oocyte ageing on the mRNA levels of the selected transcripts in common carp (mean ± SD).

The mRNA levels of 16 selected transcripts were quantified in freshly ovulated eggs and in aged eggs at different HPOs using *gapdh* as the reference gene. Genes related to the oxidative injury and stress response (*hsp70*, *sod* and *gpx1*) did not show any significant changes in their mRNA abundance during *in vivo* oocyte ageing (Fig 3). The increased level of *cox1*, however, was observed at 10–12 HPO. Among the genes related to the cell cycle, the mRNA levels of *cyclinA* and *jnkA* increased during oocyte ageing, while *cyclinB* and *jnkB* exhibited constant levels. Relative mRNA abundance of the apoptosis related genes (*caspase3A*, *caspase9* and bax) increased during oocyte ageing while the relative abundance of *bcl2*, *cathepsinB* and *cathepsinZ* remained constant during this period. Both *vasa* and *igf2* showed a constant levels during post-ovulatory oocyte ageing.

#### *In vitro* oocyte ageing

**Fig 4:**
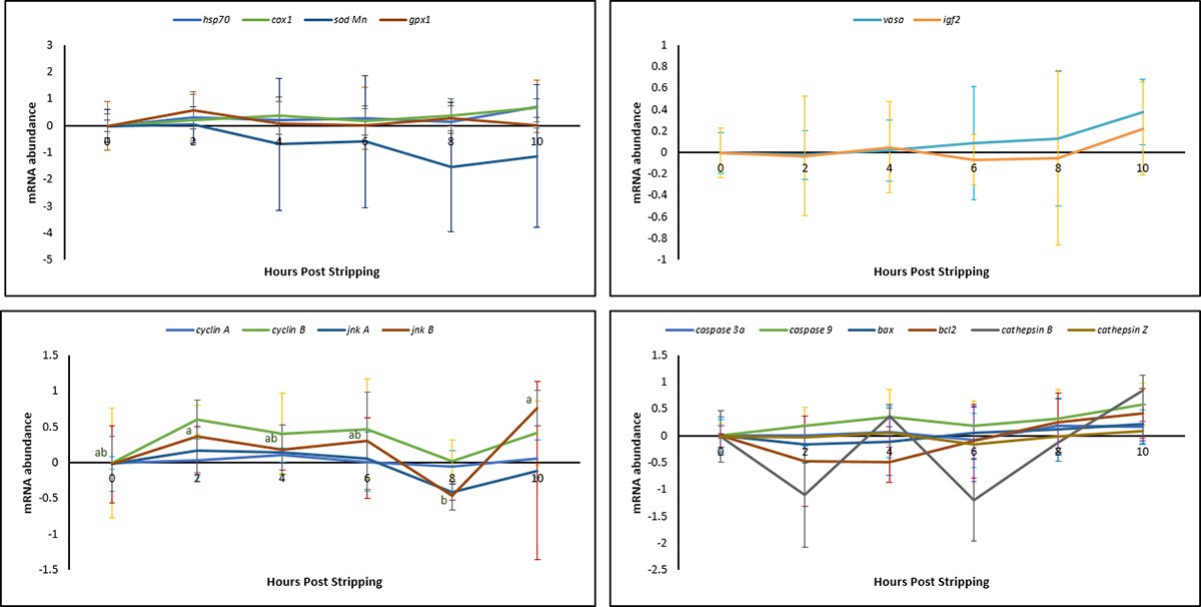
Effect of *in vitro* oocyte ageing on the mRNA levels of the selected transcripts in common carp (mean ± SD).

The mRNA profiles of the same selected genes were examined during 10 HPS. Except for *jnkB*, none of the examined genes showed any significant changes in their mRNA abundance during *in vitro* oocyte ageing for 10 hours after the stripping (Fig 4).

### Effect of oocyte ageing on the lipid and protein oxidation of the oocytes

#### *In vivo* oocyte ageing

**Fig 5:**
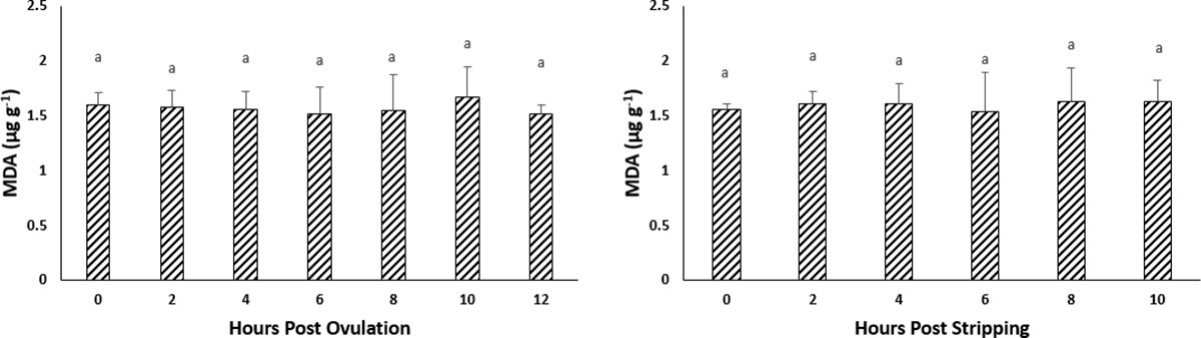
Effects of *in vivo* and *in vitro* oocyte ageing in common carp on TBARS, expressed as malonaldehyde (MDA) (μg g^-1^) (mean ± SD).

**Fig 6:**
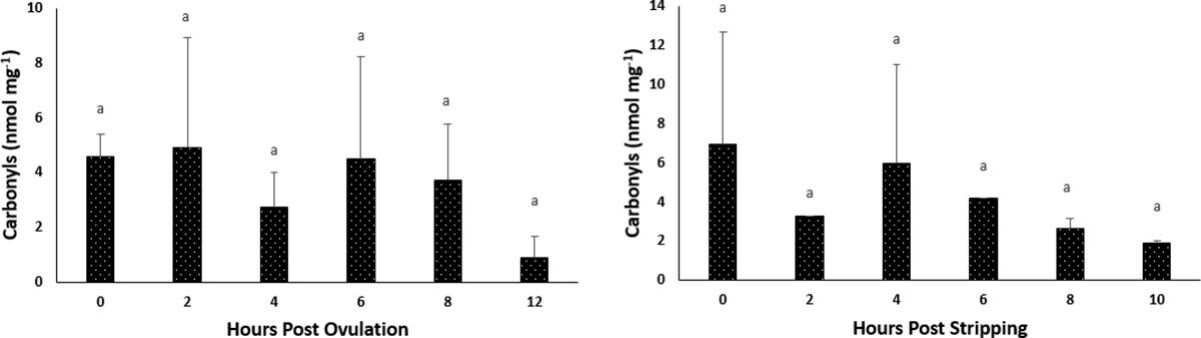
Effects of *in vivo* and *in vitro* oocyte ageing in common carp on carbonyls (nmol mg^-1^) (mean ± SD).

The level of TBARS, an intracellular stress marker, in the oocytes exhibited no significant changes during post-ovulatory ageing, remaining at approximately 1.5 MDA (μg g^-1^) (Fig 5). Protein oxidation, measured as carbonyl values, also did not show any significant changes through 12–14 HPO (Fig 6).

#### *In vitro* oocyte ageing

The level of TBARS did not show any significant changes during post-stripping oocyte ageing (Fig 5). Similarly, carbonyl values, also stayed constant through 10 HPS (Fig 6).

Comparing the *in vivo* and *in vitro* egg storage results showed no significant changes for any of the examined genes at any of the time points except for the *cathepsinZ*, *cyclinA* and *jnkA* at 4–6 hours.

### Effect of oocyte ageing on the activity of antioxidant enzymes

#### *In vivo* oocyte ageing

**Fig 7:**
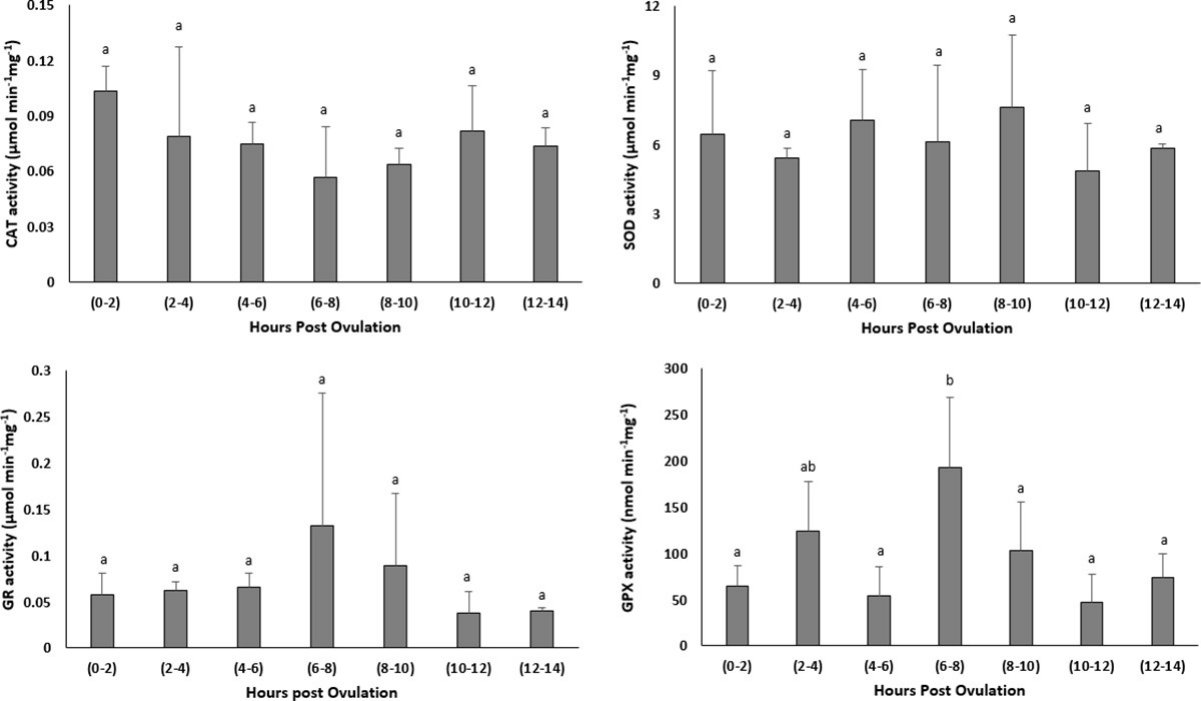
Effects of *in vivo* oocyte ageing in common carp on the activities of catalase (CAT) (μmol/min/mg), superoxide dismutase (SOD) (μmol/min/mg), glutathione reductase (GR) (μmol/min/mg) and glutathione peroxidase (GPX) (μmol/min/mg) (mean ± SD).

The activity of antioxidant enzymes, CAT, SOD and GR remained stable with prolonged *in vivo* storage of the oocytes (Fig 7). The activity of GPX however, increased significantly up to 6–8 HPO.

#### *In vitro* oocyte ageing

**Fig 8:**
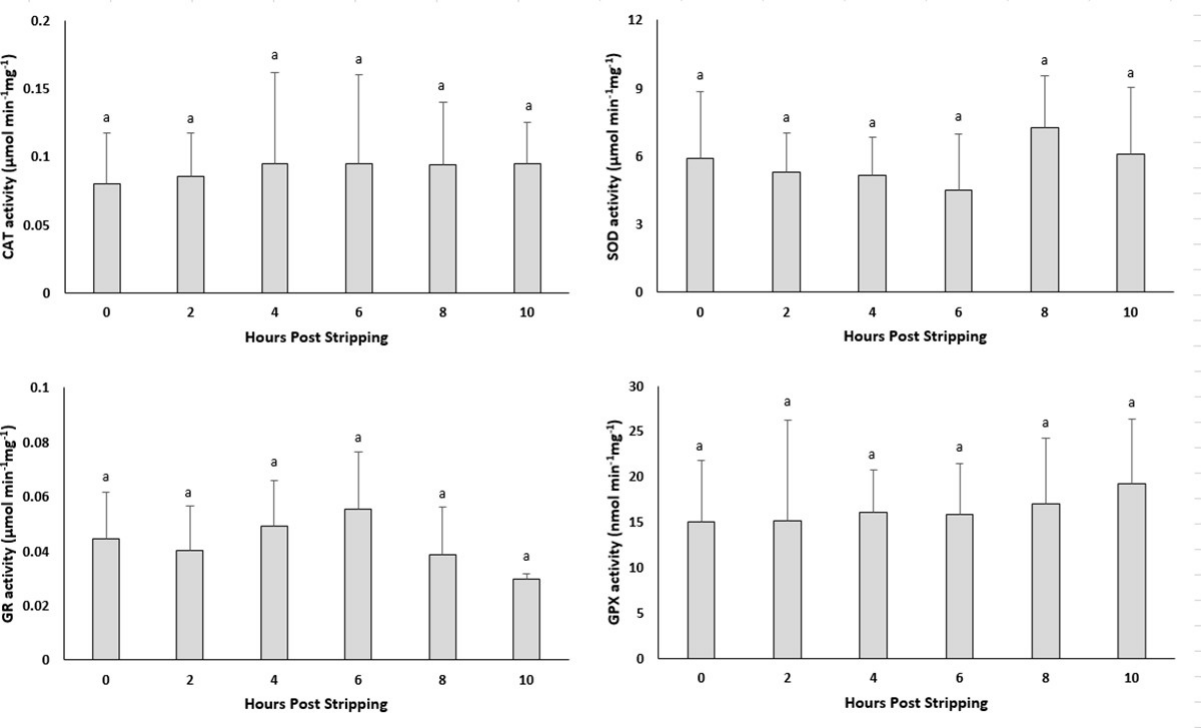
Effects of *in vitro* oocyte ageing in common carp on the activities of catalase (CAT) (μmol/min/mg), superoxide dismutase (SOD) (μmol/min/mg), glutathione reductase (GR) (μmol/min/mg) and glutathione peroxidase (GPX) (μmol/min/mg) (mean ± SD).

The CAT, SOD, GR and GPX activities did not show any significant changes through post-stripping oocyte ageing (Fig 8).

## Discussion

With elapsing time after ovulation, oocytes experience changes that negatively affect the egg quality and successive developmental stages. In the present study, the egg quality remained constant when they were stored up to 4–6 hours *in vivo* and 6 hours *in vitro* after ovulation at 20°C. The observed decreasing trend for the eyeing and hatching rates and the increasing trend for the eyed-egg mortality and larval malformation percentages after ovulation is in accordance with the previously reported experiments on the other fish species [10, 16, 18]. However, the successful egg storage time differs from a few minutes to a few weeks and highly depends on the fish species and the storage temperature [9].

It is currently undetermined as to whether a single event acts to trigger a cascade of other factors or if several biochemical and functional changes occur separately to create an aged oocyte [45]. Most authors believe that the onset of ageing in oocytes is associated with an increase in ROS, consequently leading to an increase in oxidative stress [23–25, 45]. The important consequences of oxidative stress in the oocytes are ROS-induced mitochondrial dysfunction, lipid alterations and DNA fragmentation, followed by decreasing ATP production and apoptosis. Esponda and Diaz, 2006 [46] analysed the presence of *hsp70* mRNA and protein in samples collected from 20 HPO aged mice oocytes and found that Hsp70 protein is not present in freshly ovulated oocytes; however, this protein appears in the cytoplasm of aged oocytes. They also reported increased *hsp70* mRNA levels in the aged oocytes. The intra-cytoplasmic level of glutathione, which has a major role in protecting oocytes from damage by ROS, decreased in aged mouse oocytes [47]. Additionally, the level of lipid peroxidation, which is an indicator of the degree of oxidative stress, increased in the *in vivo*-aged mouse oocytes [24]. In human embryos, a positive correlation has been reported between the concentration of hydrogen peroxide and the occurrence of apoptosis [48], indicating the initiating role of hydrogen peroxide on ageing. Hence, we examined the idea proposed for other vertebrates of whether oxidative stress affects the progress of oocyte ageing. Our results demonstrated no significant changes in the mRNA levels of oxidative stress-related genes or genes involved in the cell cycle during the progress of oocyte ageing in the common carp, with the exception of *cox1*. Additionally, as time elapsed following ovulation, the amount of TBARs (which is the main indicator of lipid peroxidation) and the amount of carbonyls (which show the extension of protein oxidation) did not change in the oocytes, indicating no increase in oxidative stress. Our experiments regarding evaluation the activity of antioxidant enzymes during oocyte ageing have also confirmed that oxidative stress is not likely the main initiator in the progress of oocyte ageing. The enzymatic antioxidant system can scavenge ROS and therefore decrease the effect of oxidative stress. If oxidative stress would be the initiator of deleterious effects during post-ovulatory oocyte ageing, then an alteration in antioxidant enzyme activity and oxidation markers should occur following ovulation. Our results indicated no significant changes in the activity of CAT, SOD and GPX during post-ovulatory ageing of common carp oocytes. The up-regulation of *cox* was also reported in the mouse oocyte with maternal ageing, i.e., the ovarian ageing [32]. On the other hand, *cox1*, located in the mitochondrial membrane, is the last enzyme in the respiratory electron transport chain. Therefore, any changes in the mRNA expression pattern of *cox1* might affect ATP synthesis and promote mitochondrial disfunction. Hamatani et al. [32] observed a decrease in the transcription levels of ATP-related genes in mouse oocytes during maternal ageing.

The relative mRNA levels of *vasa* in our study showed an upward trend during both *in vivo* and *in vitro* oocyte ageing, however, did not differ significantly. This result is in consistent with the results obtained in another recent experiment by our research group indicating that levels of *vasa* mRNA increase during *in vitro* oocyte ageing in African catfish *Clarias gariepinus* [49]. *Vasa* is a gene involved in the development of primordial germ cells (PGCs), and its activity is required for both differentiation of the germ cells into gametes [50] and the functionality of germ cells [51]. Loss of *vasa* function in the mouse affects differentiation of the male germ cells, resulting in male sterility and lack of any phenotype [50]. Vasa protein is an essential component of germplasm and represents a poorly understood complex of RNA and proteins that is required for germ cell determination. Null mutation leads to sterility in female mice, resulting from severe defects in oogenesis [52]. On the other hand, Tarin et al. [23] concluded that oocyte ageing was associated with a distorted secondary sex ratio in favour of males. Our preliminary results with zebrafish *Danio rerio* (vasa GFP transgenic strain), indicated that oocyte ageing significantly affects the number and development of the primordial germ cells (PGCs). Since depletion of PGCs converts the sex differentiation in favour of males in zebrafish [53] and other fish species [54], the oocyte ageing may bias sex ratio in favour of males or increase the probability of the occurrence of completely sterile individuals. The latter idea can be addressed in future studies.

mRNA levels of transcripts involved in the cell cycle was also investigated upon post-ovulatory and post-stripping oocyte ageing. *CyclinA* and *jnkA* mRNAs displayed higher abundance in more aged oocytes *in vivo*, while lower levels in more aged oocytes *in vitro*. A similar increasing trend in the abundance of the *cyclinA1*, *cyclinA2* and *JNK1* has been reported in rainbow trout oocytes aged *in vivo* [19]. Similarly decreased mRNA levels of two critical cell cycle-related genes, maturation promoting factor (*MPF*) and mitogen-activated protein kinases (*MAPKs*), have been reported in oocytes aged *in vitro* in porcine [30, 55] and murine models [56]. The latter study indicated the role of critical cell cycle factors and cytoplasmic changes in spontaneous activation of the oocyte ageing. The observed opposite trend towards the increased and decreased mRNA levels of the aforementioned genes during ova ageing *in vivo* and *in vitro* is of interest for future studies.

Apoptotic cell death is the end point of the oocyte ageing process and occurs through caspase activation [57], increased levels of apoptotic signalling protein *Bax*, decreased levels of anti-apoptotic protein *Bcl-xL* [58] and DNA damage [59]. Similarly, in the current study, the genes involved in apoptosis, such as *caspase3A*, *caspase9* and *bax*, exhibited lower levels at the time of ovulation (0 HPO) than those in over-ripened eggs at 10–12 HPO. This was the same case in the *in vitro* storage; when the mRNA of the abovementioned genes showed lower abundance at 0 HPS and higher abundance in the more aged oocytes at 10 HPS. By contrast, it has been shown that the level of *bax* remains unchanged in the mouse oocytes aged *in vitro*. [60, 61]. At the end of the oocyte ageing time, pro-apoptotic molecules, such as *bax*, induce the release of *cytochrome c*, which activates caspases, while anti-apoptotic molecules, such as *bcl2*, prevent this release [62]. As the expression of the anti-apoptotic protein Bcl2 is decreased during oocyte ageing in mice [61] and pigs [55], the oocytes and developing embryos are more prone to undergo apoptosis [25, 60]. In the present study, the relative mRNA abundance of *bcl2* showed no significant changes during both *in vivo* and *in vitro* oocyte ageing. Bcl2 is known as a family of proteins regulating cell death by either inducing or inhibiting apoptosis [63, 64]. The observed trend for the relative mRNA abundance of *bcl2* in our study might be attributed to the inducing role of the examined gene for apoptosis during ova ageing, while many other genes encoding the Bcl2 protein might have an inhibiting role in the occurrence of apoptosis. Levels of *hsp70* and *cox1* mRNAs showed the increasing tendency at the beginning phase of oocyte ageing, while increased levels of *caspase3A*, *caspase9* and *bax*, were obvious at 10–12 HPO and HPS. Consistent with these observations, Lord et al. [25] suggested that oxidative stress in aged oocytes can be considered as an early marker of oocyte ageing before the activation of caspase-3 and before the appearance of the morphological features of oocyte ageing and apoptosis. Although they do not differ statistically, levels of *CathepsinB* and *cathepsinZ* mRNAs were both increased during the *in vivo* and *in vitro* egg storage, except for the initial decrease of *cathepsinZ* once the eggs stored *in vitro*. Upregulation of *cathepsinB* is associated with cell death [65]. Aegerter et al. [19] also found that *in vivo* oocyte ageing in rainbow trout is associated with the increased mRNA abundance of *cathepsinZ*. Lysosomal proteases *cathepsinD* and *cathepsinB* act as pro-apoptotic mediators of apoptosis [66]. Therefore, the increased mRNA levels of the *cathepsinB* and *cathepsinZ* genes during the oocyte ageing progress might lead to the increased mRNA levels of the apoptotic genes observed in this study.

Our results indicated that the relative mRNA level of *igf2* showed a decreasing trend to 6 HPO and HPS and then increased until the complete loss of egg viability occurred. Aegerter et al. [19] also found higher quantities of *igf2* mRNAs in more aged rainbow trout oocytes at 14 HPO than that in the freshly ovulated ones. The IGF axis has been shown to play a role in the inhibition of apoptotic cell death [67]. The increased tendency towards the mRNA levels of the *igf2* observed in this study therefore, might be considered as a defence mechanism against the occurrence of over-ripening of the eggs and the apoptosis. The same trend was observed for the mRNA abundance changes during *in vivo* and *in vitro* oocyte ageing, shows that the oocyte ageing may pass through the same processes either the eggs are aged *in vivo* or *in vitro*.

Although examining the mRNA abundance of single target genes could be a good and helpful tool, it is not yet enough to conclude on the possible involvement of oxidative stress in the progress of fish oocyte ageing. In fact, additional analysis such as microarray analysis, total ROS measurement, mitochondrial dysfunction indicators, ATP content of the eggs, etc., are required to fully evaluate the contribution of oxidative stress to the drop of egg quality during postovulatory ageing. Additionally, as there is no clear link between mRNA abundance and protein synthesis in metaphase 2 oocytes, studying the proteome profile changes during the fish oocyte ageing could provide valuable information about the oocyte ageing and its underlying mechanisms. Further analysis of these genes during development in eggs at varying ageing times will be useful and will benefit to the study on fish egg quality.

The epigenetic changes in mouse oocytes have been associated with post-ovulatory ageing [68, 69]. The ageing of oocytes has been shown to significantly alter the methylation pattern of imprinted genes in both mouse oocyte and the developing placenta [69, 70]. This process in turn alters the demethylation events after fertilization [71]. The epigenetic modification related factors (DNA methylation and histone modification) might be involved in defects arising in aged oocytes and the originating embryos. Investigating the epigenetic changes associated with fish oocyte ageing seems to be interesting for the future studies.

The results obtained in our study demonstrate that oxidative stress is not likely the main initiator of the oocyte ageing in common carp. However, complementary tests and analysis are required to clarify the involvement of oxidative injury and stress response in the progress of oocyte ageing. The apoptosis-related genes however, were significantly altered following the prolonged time interval between ovulation and fertilization demonstrating that apoptotic pathway might be involved in the progress of oocyte ageing.

## Supporting information

S1 Table Egg viability (eyeing, hatching, eyed-egg mortality and larval malformation) rates during *in vivo* oocyte ageing in common carp. (XLSX)

S2 Table Egg viability (eyeing, hatching, eyed-egg mortality and larval malformation) rates during *in vitro* oocyte ageing in common carp. (XLSX)

S3 Table mRNA abundance of the selected transcripts relative to *gapdh* during *in vivo* oocyte ageing in common carp. (XLSX)

S4 Table mRNA abundance of the selected transcripts relative to *gapdh* during *in vitro* oocyte ageing in common carp. (XLSX)

S5 Table TBARS values, expressed as malonaldehyde (MDA) (μg g^-1^) during the *in vivo* and *in vitro* oocyte ageing in common carp. (XLSX)

S6 Table Carbonyls values (nmol mg^-1^) during the *in vivo* and *in vitro* oocyte ageing in common carp. (XLSX)

S7 Table Activities of catalase (CAT) (μmol/min/mg), superoxide dismutase (SOD) (μmol/min/mg), glutathione reductase (GR) (μmol/min/mg) and glutathione peroxidase (GPX) (μmol/min/mg) during *in vivo* oocyte ageing in common carp. (XLSX)

S8 Table Activities of catalase (CAT) (μmol/min/mg), superoxide dismutase (SOD) (μmol/min/mg), glutathione reductase (GR) (μmol/min/mg) and glutathione peroxidase (GPX) (μmol/min/mg) during *in vitro* oocyte ageing in common carp. (XLSX)
